# Antenatal maternal intimate partner violence exposure is associated with sex-specific alterations in brain structure among young infants: Evidence from a South African birth cohort

**DOI:** 10.1016/j.dcn.2023.101210

**Published:** 2023-02-06

**Authors:** Lucy V. Hiscox, Graeme Fairchild, Kirsten A. Donald, Nynke A. Groenewold, Nastassja Koen, Annerine Roos, Katherine L. Narr, Marina Lawrence, Nadia Hoffman, Catherine J. Wedderburn, Whitney Barnett, Heather J. Zar, Dan J. Stein, Sarah L. Halligan

**Affiliations:** a Department of Psychology, University of Bath, Bath, UK; b Department of Pediatrics and Child Health, Red Cross War Memorial Children’s Hospital and University of Cape Town, Cape Town, South Africa; c The Neuroscience institute, University of Cape Town, Cape Town, South Africa; d SAMRC Unit on Risk & Resilience in Mental Disorders, Department of Psychiatry and Mental Health, University of Cape Town, Cape Town, South Africa; e Department of Psychiatry and Mental Health, University of Cape Town, Cape Town, South Africa; f Departments of Neurology, Psychiatry and Biobehavioral Sciences, University of California, Los Angeles, CA, USA; g Department of Clinical Research, London School of Hygiene & Tropical Medicine, London, UK; h Department of Psychology and Human Development, Vanderbilt University, USA; i SAMRC Unit on Child & Adolescent Health, University of Cape Town, Cape Town, South Africa; j Department of Psychiatry, Stellenbosch University, Stellenbosch, South Africa

**Keywords:** Antenatal stress, Intimate partner violence, Neonate, Brain imaging, LMIC, Trauma

## Abstract

Maternal psychological distress during pregnancy has been linked to adverse outcomes in children with evidence of sex-specific effects on brain development. Here, we investigated whether *in utero* exposure to intimate partner violence (IPV), a particularly severe maternal stressor, is associated with brain structure in young infants from a South African birth cohort. Exposure to IPV during pregnancy was measured in 143 mothers at 28–32 weeks’ gestation and infants underwent structural and diffusion magnetic resonance imaging (mean age 3 weeks). Subcortical volumetric estimates were compared between IPV-exposed (*n* = 63; 52% female) and unexposed infants (*n* = 80; 48% female), with white matter microstructure also examined in a subsample (IPV-exposed, *n* = 28, 54% female; unexposed infants, *n* = 42, 40% female). In confound adjusted analyses, maternal IPV exposure was associated with sexually dimorphic effects in brain volumes: IPV exposure predicted a larger caudate nucleus among males but not females, and smaller amygdala among females but not males. Diffusivity alterations within white matter tracts of interest were evident in males, but not females exposed to IPV. Results were robust to the removal of mother-infant pairs with pregnancy complications. Further research is required to understand how these early alterations are linked to the sex-bias in neuropsychiatric outcomes later observed in IPV-exposed children.

## 1 Introduction

Maternal psychological distress during pregnancy is associated with an increased risk of mental health problems in children (Davis and Sandman, 2012, Park et al., 2014) and predicts psychiatric disorders in adulthood (Betts et al., 2015, Van den Bergh et al., 2020). The experimental induction of prenatal stress in animal models has shown enduring effects on neurodevelopment of the offspring (Soares-Cunha et al., 2018, Zhang et al., 2021). Evidence points to how the excessive release of cortisol, serotonin (Bush et al., 2017, Sandman et al., 2011) or pro-inflammatory cytokines (Glover, 2015) may be one of numerous mediating factors that lead to alterations in neurochemistry and signalling pathways involved in regulating neuroplasticity (Lai and Huang, 2011, Lautarescu et al., 2020). Limbic brain regions may be particularly sensitive to prenatal stress due to their high content of glucocorticoid receptors (Teicher et al., 2003), while animal models also indicate sex-specific biological responses to prenatal stress (Gabory et al., 2009, Mueller and Bale, 2008) that may lead to different behavioural phenotypes (Glover and Hill, 2012, Weinstock, 2017).

Psychological distress in humans during pregnancy encompasses symptoms of anxiety, depression, as well as the effects of exposure to stressful or traumatic life events including intimate partner violence, bereavement, or natural disasters (Wu et al., 2020). The adverse outcomes observed in children later in life is presumably due to how psychological distress affects brain structure and biochemistry *in utero*. Brain imaging studies have assessed the relationship between variations in cortisol as a marker of antenatal maternal psychological distress and the subsequent development of limbic brain structures in children and adolescents many years later. Consistent with the preclinical literature, most evidence points to how antenatal maternal psychological distress may influence the offspring amygdala in a sex-specific manner, with likely indicators of antenatal maternal distress being associated with enlarged amygdala volumes in school-aged girls, but not boys (Acosta et al., 2019, Buss et al., 2012, Jones et al., 2019, Wen et al., 2017), albeit with some inconsistent findings (El Marroun et al. (2016). For example, higher maternal cortisol measured in pregnancy predicted larger right amygdala volumes in 7-year-old girls, which mediated later affective problems (Buss et al., 2012). Additionally, female children of mothers exposed to a natural disaster during pregnancy showed larger amygdala volumes at aged 11, which in part explained greater externalizing problems (Jones et al., 2019). In contrast, evidence for changes to the hippocampus due to prenatal stress has been mixed. While reduced hippocampal volume is evident in adults and children exposed to *early life stress* (Humphreys et al., 2019, Marečková et al., 2018, Woon and Hedges, 2008), there is little evidence for an association between hippocampal volume and *antenatal* stress in humans (Buss et al., 2012, Favaro et al., 2015, Marečková et al., 2018, Qiu et al., 2013). Importantly, few studies have examined the developing brain in very young infants to minimize the impact of the postnatal environment as, for example, parenting styles may also contribute to children’s brain development (Suffren et al., 2022). One recent study reported no association between maternal cortisol concentrations measured in pregnancy and neonatal amygdala volume (age range: 39–46 weeks’ gestation), but found evidence that high cortisol exposure in girls was associated with increased structural connectivity between the amygdala and other brain regions (Stoye et al., 2020). However, normative maternal salivary cortisol levels are unrelated to self-reported psychological measures of distress (Voegtline et al., 2013) and may not capture the same effects elicited by exposure to more severe stressors.

Prenatal exposure to maternal psychological stress has also been linked to the disruption of the microstructural organization of white matter tracts in offspring, particularly within amygdala-prefrontal circuits given the importance of this circuity in emotion regulation (Costanzo et al., 2016, Von Der Heide et al., 2013). Diffusion tensor imaging (DTI) studies have linked prenatal stress during pregnancy with greater mean diffusivity (MD) in the uncinate fasciculus in both school-aged children (El Marroun et al., 2018) and in pre-term neonates (Lautarescu, Pecheva et al., 2020). Increases in diffusivity suggests the reduced integrity of neural barriers to the free diffusion of water and typically indicate a loss of organized structure (Alexander et al., 2007). Furthermore, prenatal stress, as indexed by higher maternal anxiety, predicted *reduced* fractional anisotropy (FA) in full-term neonates within the uncinate fasciculus, among several other regions essential to socio-emotional function (posterior cingulate, parahippocampus) and cognitive-emotional responses to stress (insula and dorsolateral prefrontal cortex) (Rifkin-Graboi et al., 2015). FA, a measure indicating the overall directionality of water diffusion, is typically greater in organized white matter tracts and lower in CSF and crossing fibres, and reflects the relative alignment of individual axons, how tightly they are packed (which affects the amount of interstitial water), myelin content, and axon diameter (Alexander et al., 2007). Further studies have begun to consider the effects of prenatal maternal distress on the microstructural organization of other white matter tracts. For example, exposure to prenatal stress has been associated with disruptions to the anterior cingulate (i.e. cingulum) within 2–5 weeks after birth (Demers et al., 2021), and corpus callosum in 6-month-old infants (Borchers et al., 2021). Given this emerging evidence, the potential impacts of prenatal stress on white matter pathways in young infants, including possible sex-specific effects, warrants further investigation.

One particularly severe stressor that can occur antenatally is exposure to intimate partner violence (IPV) (Barnett et al., 2018). According to large population-based household and national health surveys, the prevalence of violence against women by their intimate partner varies widely across countries but tends to be substantially higher in low-income and middle-income countries versus high-income countries (Coll et al., 2020, Garcia-Moreno et al., 2006). Maternal IPV exposure during pregnancy not only has negative mental and physical health consequences for women (McKelvie et al., 2021), but is also associated with poor health and developmental outcomes for their children (Chai et al., 2016, Da Thi Tran et al., 2022) and is a serious public health concern. The present study utilizes data from the Drakenstein Child Health Study (DCHS), a multidisciplinary South African birth cohort study, to conduct the first examination of how antenatal IPV exposure affects early brain development. Based on previous DCHS findings which found an association between antenatal depression exposure and infant brain structure (Groenewold et al., 2022), we hypothesized that exposure to antenatal IPV would have a similar impact on neurodevelopment. Our primary objective was to establish whether maternal IPV exposure during pregnancy is associated with alterations in neonatal subcortical brain volumes and white matter microstructure in offspring within the first few weeks of life, including the testing of sex-specific effects. Specifically, we examined IPV in relation to volumes of the amygdala, hippocampus, and other key subcortical regions in the basal ganglia and thalamus in neonates. In a subset of the sample, we also examined microstructural white matter integrity, measured by FA and MD, of limbic white matter tracts implicated in the development of psychopathology in older children (uncinate fasciculus, cingulum, and fornix) as well as tracts previously found to be sensitive to the deleterious effects of stress (corpus callosum and corticospinal tract).

## 2 Materials and methods

### 2.1 Study population

**Fig. 1:**
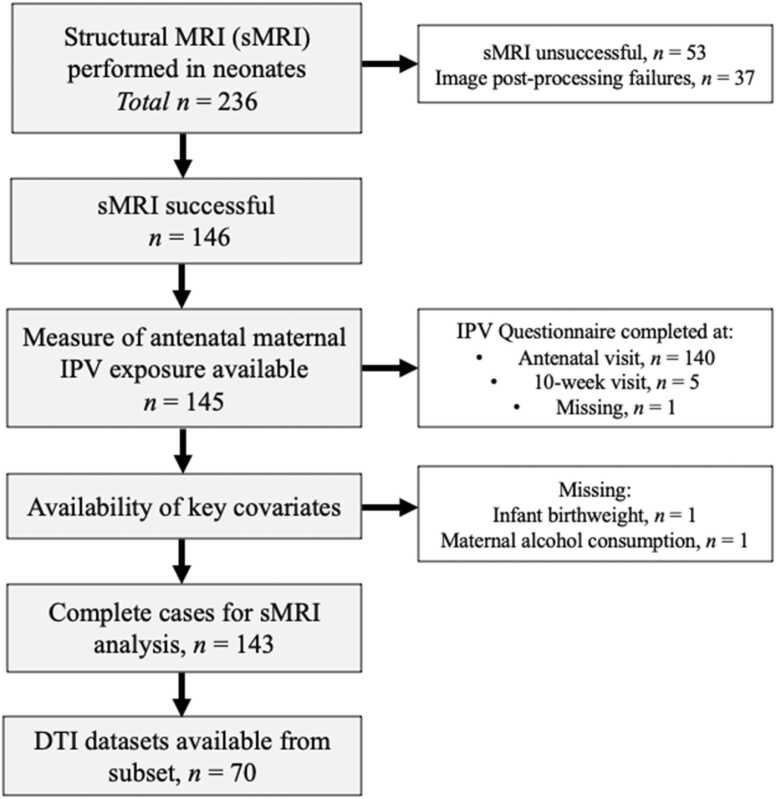
Drakenstein Child Health Study cohort flow chart of neonates with neuroimaging. Structural MRI was unsuccessful in infants who did not sleep or because of artefacts evident during the scan. In the 183 infants with eligible T_2_-weighted images, 37 did not pass quality control either due to poor image quality, normalization errors, or segmentation faults leaving a total of 146 infants with structural MRI data available. We were unable to account for missing birthweight (*n* = 1) and missing antenatal alcohol consumption (*n* = 1), as they are considered exogenous thus are not predicted by any of the other variables in the model.

The Drakenstein Child Health Study (DCHS) is a multi-disciplinary longitudinal birth cohort investigating the early-life determinants of child health in two impoverished communities located in the Western Cape Province of South Africa (Stein et al., 2015, Zar et al., 2015). The full cohort consisted of 1137 expectant mothers in their second trimester who were recruited from two public primary health care clinics: Mbekweni (serving a Black African community) and TC Newman (serving a mixed-ancestry community). Between September 2012 and September 2015, 236 of these mother-infant pairs (20.6% of full cohort) were invited to take part in a brain magnetic resonance imaging (MRI) sub-study approximately 2–6 weeks after birth (Fig. 1). Infants were excluded if they had: (1) any medical comorbidities, including a genetic syndrome, neurological disorder or congenital abnormality; (2) low Apgar score (<7 at 5 min); (3) neonatal intensive care admission; (4) maternal use of illicit drugs during pregnancy; (5) MRI contraindications; or (6) infant HIV infection. Previous studies have demonstrated a high burden of poverty-related stressors during pregnancy in this cohort including substance use, low education, and depressive symptoms (Groenewold et al., 2022, Stein et al., 2015).

All protocols received ethical approval from the University of Cape Town, Faculty of Health Sciences, Human Research Ethics Committee (full cohort: 401/2009; MRI sub-study 525/2012). The parent study was approved by the Western Cape Provincial Health Research committee (2011RP45). All study procedures were carried out in accordance with the Declaration of Helsinki (World Medical Association, 2013).

### 2.2 Measures and variable calculation

Questionnaires were administered to the enrolled women at the primary health care clinics between 28- and 32-weeks’ gestation. The questionnaires were offered in locally spoken languages in the Western Cape (English, Afrikaans, and Xhosa). Approximately 50% of questionnaires were administered in Afrikaans, whereas the others were administered in either English (∼25%) or Xhosa (∼25%).

#### 2.2.1 Intimate partner violence exposure

The Intimate Partner Violence (IPV) Questionnaire assessed mothers’ recent exposure (past 12 months) to emotional, physical, and sexual abuse, and has been adapted from the WHO multi-country study on women’s health and domestic violence against women (WHO, 2005). There are four questions relating to emotional violence (e.g., being purposefully scared or intimidated), five relating to physical abuse (e.g., being hit with a fist or with something else that could hurt), and three questions assessing sexual violence (e.g., being physically forced to have sex). A 4-point frequency of occurrence scale was used for each of the 12 questions: (1) never, (2) once, (3) few times, and (4) many times, resulting in a maximum score of 48. At the end of the emotional violence questions, mothers were then asked, *“Have any of these things happened in the past 12 months?”.* The same question was asked again at the end of both the physical and sexual violence questions. If the answer was “*Yes”* to any one of the three violence categories (emotional, physical, or sexual), mothers were classified as being exposed to recent IPV. Participants were designated into the control group if they were not exposed to any type of IPV within the previous 12 months. Therefore, antenatal exposure to IPV was used as a dichotomous variable (exposure to recent IPV/ no exposure to recent IPV) in all statistical models.

#### 2.2.2 Demographic and clinical variables

*Sociodemographic:* Maternal sociodemographic characteristics were collected by interview and questionnaires, including mother’s age, employment status, marital status, household income, maternal education, as well as medical history including HIV status.

*Birth characteristics:* Gestational age at birth (in weeks) was recorded either through ultrasound, measurements of fundal height, or through self-reported last menstrual period. Birth weight was obtained at the hospital following delivery.

*Prenatal alcohol consumption and smoking status:* Prenatal alcohol exposure risk was classified as a binary variable, defined as an Alcohol, Smoking and Substance Involvement Screening Test (ASSIST) total alcohol score > 10 (at least weekly alcohol use with negative consequences) or retrospective self-report of consuming alcohol on 2 or more occasions per week (Newcombe et al., 2005). Smoking status was assessed via prenatal cotinine measurements in maternal urine using the IMMULITE 1000 Nicotine Metabolite Kit (Siemens Medical Solutions Diagnostics, Glyn Rhonwy, Llanberis, UK). Cotinine is the major metabolite of nicotine and is often used as a proxy measure of recent tobacco use. Active smoking was defined by cotinine > = 500 ng/ml, with passive smoking > = 10–499 ng/ml, and nonsmoking < 10 ng/ml.

*Maternal depression*: Antenatal maternal depressive symptoms were assessed using the Beck Depression Inventory II (BDI-II; Beck et al. (1996), which has been validated and used in multiple studies of South African women (Kagee et al., 2014). The measure consists of 21 items, each assessing major depressive symptoms over the past four weeks (rated 0–3, total score range 0–63). A cut off score of ≥ 20 was used for dichotomizing participants into “probable moderate/severe clinical cases” versus “sub-threshold participants”, as previously described Stein et al. (2015).

### 2.3 Image acquisition

MR images were acquired on a 3 T Siemens Magnetom 3 T Allegra MRI scanner (Allegra MR2004A, Germany) at the Cape Universities Brain Imaging Centre (CUBIC), Tygerberg Hospital, Cape Town. Infants were fed, swaddled in a blanket, and encouraged to sleep. Earplugs and mini muffs were used for double ear protection and a qualified neonatal nurse or pediatrician monitored the infant in the scanner room for the duration of the scan (Wedderburn et al., 2022). To overcome limitations with scanning smaller tissue volumes, voltage was reduced to optimize signal, and a radiofrequency transmit/receive head coil was loaded with a wet clay inlay (40x40cm, 2 cm thickness, standard sculpting clay). The imaging protocol included a *T*_2_-weighted anatomical scan with the following parameters: FOV = 160 × 160 mm, *TR* = 3500 *ms*, *TE* = 354 *ms*, 128 slices, in-plane resolution = 1.3 × 1.3 mm and a slice thickness of 1.0 mm. The acquisition time was 5 min 41 s. Diffusion weighted images were collected using a spin-echo echo-planar imaging (EPI) sequence with images collected in both the anterior–posterior (AP) and posterior–anterior (PA) phase encoding directions to correct for field inhomogeneities. Parameters were as follows: 30 diffusion directions; FOV = 160 × 160 mm, *TR* = 7800 *ms*, *TE* = 91 *ms*, voxel size 1.8 × 1.8 × 2.0 mm^3^; *b*-value 1 of 0 s/mm^2^ and b-value 2 of 1000 s/mm^2^. Total diffusion scan time was 12 mins 54 s

### 2.4 Image processing

From the original sample of 236 infants, 53 infants did not sleep or were excluded due to artefacts leaving a sample of 183 infants (78%) for image processing. T_2_-weighted images were skull stripped using the brain extraction tool (BET) from the FMRIB Software Library (FSL) v5.0 (Jenkinson et al., 2012). Each scan was checked visually following the initial BET and additional custom thresholds were applied to ensure non-brain tissue was adequately removed. Brain images were pre-processed further using Statistical Parametric Mapping software (SPM8, University College London, London, UK) with researchers blinded to IPV exposure status. Images were first registered and then spatially normalized to the University of North Carolina custom infant T_*2*_ template in Montreal Neurological Institute (MNI) space (Shi et al., 2011). Normalized images were segmented into grey matter, white matter, and cerebrospinal fluid in accordance with the corresponding neonate probabilistic maps. Alignment to the template and segmentation accuracy was confirmed through visual inspection (see Supplementary Methods for more details). From the 183 infants with eligible T_2_-weighted images, 37 did not pass quality control either due to poor scan quality (*n* = 18), normalization errors (*n* = 4), segmentation faults (*n* = 13), or on further post-processing checks (*n* = 2), leaving a total of 146 infants with structural MRI data available. Bilateral volumes were extracted for total grey matter (GM), total white matter (WM), and subcortical grey matter structures of interest (amygdala, caudate, hippocampus, pallidum, putamen, and thalamus) as defined by the automated anatomical labeling atlas (Tzourio-Mazoyer et al., 2002).

Diffusion tensor imaging was performed in *n* = 158 infants (67%) from the original cohort at the same scanning session. A combination of technical artefacts, motion, further exclusions on pre-and post- processing checks, or data missing for the antenatal IPV questionnaire resulted in a reduced sample size of *n* = 70. The acquisition of high-quality diffusion data is a common challenge in paediatric neuroimaging (Barkovich et al., 2019) due to the imaging sequence’s greater sensitivity to movement and longer acquisition time. Images with at least 15 usable volumes with minimal movement or other artifacts were deemed usable for data pre-processing and statistical analyses (see Supplementary Methods). Usable diffusion data was preprocessed using TORTOISE v2.5.2 (Tolerably Obsessive Registration and Tensor Optimization Indolent Software Ensemble), which implements comprehensive correction and applies greater anatomical registration ability compared to mainstream diffusion processing pipelines (Taylor et al., 2016). The DiffPrep module was used to compute distortion corrections for participant motion, eddy currents and EPI distortions on each AP and PA encoded image. The DR BUDDI module merged the encoded sets and performed further EPI distortion corrections. Diffusion tensor parameter fitting was then performed with Tract-Based Spatial Statistics (TBSS) in FSL to extract parameters (Smith et al., 2006). A study-specific template was used to enhance registration quality, given the infant brain will differ from the adult MNI template. Fractional anisotropy (FA), a standardized measure of directional water diffusion, and mean diffusivity (MD), the average of water diffusion, were extracted for the five white matter tracts of interest (uncincate fasciculus, fornix, cingulum, corticospinal tract, and corpus callosum) using the Johns Hopkins University ICBM-DTI-81 atlas (Mori et al., 2008).

### 2.5 Missing data

Fig. 1 details the proportion of missing data related to potential confounding variables. Infants were included in the analysis if either structural or diffusion MRI data, recent IPV exposure, and all potential covariates were available.

### 2.6 Statistical analysis

Socio-demographic and clinical characteristics were compared between IPV-exposed and unexposed groups using independent *t*-tests (continuous variables), and chi-squared tests (categorical variables). Group differences in neuroimaging outcomes were assessed using a series of full factorial linear regression models, with each brain structure of interest as the dependent variable and recent IPV and infant sex as predictors. In the event of a significant interaction between IPV exposure and infant sex, sex-stratified analyses were conducted in boys and girls separately. A similar approach was taken for the DTI analyses. Confounders were selected *a priori* and included household income – as a proxy for socioeconomic status, maternal HIV status, maternal depression symptoms, gestation duration, infant birthweight, prenatal tobacco exposure, and prenatal alcohol exposure. The mean variance inflation factor of covariates was 1.58 - which is acceptable to meet the multicollinearity assumption. All covariates were categorical variables, except for gestation duration and birthweight which were continuous. To account for subject-specific and group-specific global differences in head size, subcortical volumes were normalized to the estimated total intracranial volume (eTIV). Corrections were performed by adjusting regional volumes using the analysis of covariance (ANCOVA) approach, which corrects regional measures based on the proportion of the difference between an individual’s global brain measure and the average global brain measure for the sample (Delgorio et al., 2022, Raz et al., 2005). The Shapiro-Wilk W test was used to test for a normal distribution, and the Cameron & Trivedi’s IM-test assessed for heteroscedasticity. If assumptions of normality were violated, Cook’s *d* identified outliers by providing an overall score of the residuals and leverage for each observation. Outliers were removed until the assumptions for normality and heteroscedasticity were met, which ranged between 0 and 3 observations across neuroimaging outcomes **(**Supplementary Table 3 **[ST3]).** Standard main effect sizes were calculated using Cohen’s *d*, whereas the effect size of the interaction term was determined by partial η^2^ with small, medium, and large effect sizes corresponding to values of 0.0099, 0.0588, and 0.1379, respectively (Cohen, 1969). Finally, sensitivity analyses were performed to assess the robustness of the results by excluding mother-infant dyads who had experienced pregnancy complications, including gestational diabetes (*n* = 1), preeclampsia (*n* = 4), pelvic inflammatory disease (*n* = 1), and/or high blood pressure (*n* = 5).

Statistical analyses were conducted using STATA, v17.0 (StataCorp Inc, College Station, TX, USA). *p* < 0.05 was used as a threshold of statistical significance. To minimize the number of comparisons, unilateral volumes were added together to create bilateral volumes, whereas DTI outcomes were averaged across hemispheres. The standard False Discovery Rate method was used as an additional correction for multiple comparisons (Benjamini and Hochberg, 1995) (*n* = 6 for subcortical volumes; *n* = 10 for diffusion outcomes) with a false-positive rate of 5% (*q* = 0.05).

## 3 Results

### 3.1 Participant characteristics for IPV-exposed and unexposed infants

The final sample for the structural MRI analysis included 143 mother-child dyads. Sixty-three mothers (44%) reported IPV within the preceding 12 months, whereas 80 mothers (56%) who did not report IPV exposure served as a control group. Mothers in the two groups were similar in most sociodemographic characteristics including age at birth, education level, household income, and employment status (Table 1). However, mothers exposed to IPV were more likely to be married or living with a partner compared to controls (53.9% versus 33.8%, *χ*^*2*^ = 6.34, *p* = 0.012), whereas the majority of the control group were single. Antenatal alcohol consumption was more prevalent in the IPV exposed compared to unexposed group (27.0% versus 11.3%, *χ*^*2*^ = 5.87, *p* = 0.015), whereas IPV-exposed mothers were less likely to be HIV positive than controls (17.5% versus 32.5%, *χ*^*2*^ = 4.16, *p* = 0.041). Two mothers were on psychotropic medication at enrolment. One IPV-exposed mother reported antidepressant medication use (citalopram), whereas one unexposed mother reported pain medication use (tramadol). There were no group differences in infant birth characteristics with regards to sex, age at scan, gestation duration, birthweight, and head circumference, and the imaging subsample was largely representative of the wider DCHS sample (Wedderburn et al., 2022). Overall, sixteen infants (11%) were moderate to late preterm (between 33 and 37 weeks gestational age at birth), whereas the majority were born at full term. All pregnancies were uniparous. From the subset of 143 mother-child dyads with structural MRI data and all key covariates available, DTI data were available from 70 infants (See Supplementary Table 2 **(ST2)** for characteristics of this subsample).

**Table 1:** Descriptive characteristics of IPV-exposed and unexposed groups.

	IPV-exposed (*n* = 63)	Controls (*n* = 80)	Group differences (*χ*^2^ or *t*)	*p*-value
**Sociodemographic**				
**Clinic (*****n*****, %)**				
*Mbekweni*	28, 44.4%	42, 52.5%	0.92	0.339
*TC Newman*	35, 55.6%	38, 47.5%		
*Maternal age at birth (M±SD)*	27.98 ± 5.72	26.92 ± 6.04	-1.06	0.290
**Household income per month (*****n*****, %)**				
*<R1000*	19, 30.2%	28, 35.0%	1.41	0.493
*R1000 – R5000*	36, 57.1%	38, 47.5%		
*>R5000*	8, 12.7%	14, 17.5%		
**Employment status (n, %)**				
*Employed*	14, 22.2%	26, 32.5%	1.85	0.174
*Unemployed*	49, 77.8%	54, 67.5%		
**Maternal education (*****n*****, %)**				
*Primary/some secondary*	36, 57.1%	41, 51.2%	0.49	0.483
*Completed secondary/any tertiary*	27, 42.9%	39, 48.8%		
**Marital status (*****n*****, %)**				
*Single (never married)*	28, 44.4%	53, 66.2%	6.34	0.012
*Married/living with partner*	34, 53.9%	27 33.8%		
**Maternal clinical characteristics**				
**HIV status (*****n*****, %)**				
*Positive*	11, 17.5%	26, 32.5%	4.16	0.041
*Negative*	52, 82.5%	54, 67.5%		
**Beck Depression Inventory (*****n*****, %)**				
*Above threshold*	21, 33.3%	20, 25.0%	1.20	0.274
*Below threshold*	42, 66.7%	60, 75.0%		
**Antenatal alcohol exposure (*****n*****, %)**				
*Exposure*	17, 27.0%	9, 11.3%	5.87	0.015
*No exposure*	46, 73.0%	71, 88.8%		
**Antenatal tobacco use (*****n*****, %)**				
*Non-smoker (<10 ng/ml)*	13, 20.6%	26, 32.5%	3.80	0.150
*Passive smoker (>=10–499 ng/ml)*	26, 41.3%	34, 42.5%		
*Active smoker (>=500 ng/ml)*	24, 38.1%	20, 25.0%		
**Infant characteristics**				
**Sex (*****n*****, %)**				
*Male*	30, 47.6%	42, 52.5%	0.576	0.448
*Female*	33, 52.4%	38, 47.5%		
Age at scan (weeks) (*M* ± *SD*)	3.22 ± 0.98	3.13 ± 0.76	-0.62	0.534
Gestation duration (weeks) (*M* ± *SD*)	38.95 ± 1.96	38.95 ± 1.91	-0.01	0.994
Birthweight (grams) (*M* ± *SD*)	3108 ± 469	3200 ± 454	1.19	0.236
Head circumference (cm) (*M* ± *SD*)	33.52 ± 1.72	33.99 ± 1.66	1.66	0.099

*Missing marital status (*n* = 1). R South African Rand. R1000 is approximately equal to US $60.

### 3.2 Effects of antenatal IPV exposure on neonatal brain volumes

**Fig. 2:**
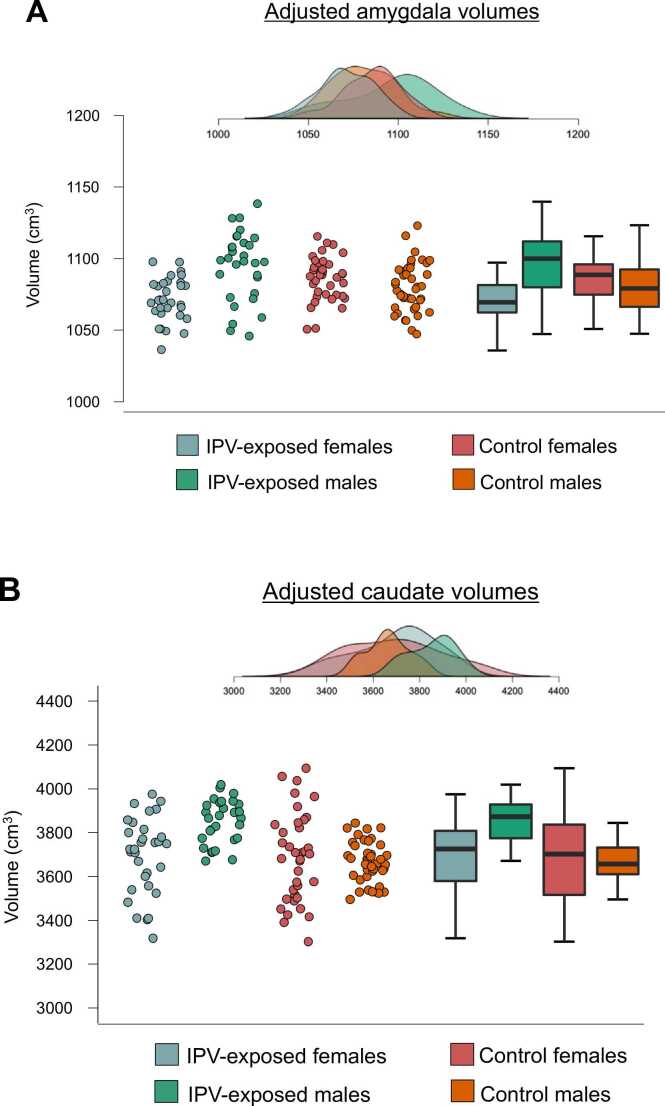
Raincloud plots (jittered data points for all participants, boxplots, and probability distribution of the data) for predicted values of (a) amygdala volume, and (b) caudate volume. For the boxplots, the boxes and the horizontal line inside show the quartiles (1st to 3rd quartile) and the median, respectively. The whiskers denote 1.5 times the interquartile range.

Given the exploratory nature of this study, all models were initially run without correction for multiple comparisons. There were no main effects of antenatal IPV exposure on total WM or GM volumes, and no IPV-by-sex interactions. However, infants who were exposed to IPV *in utero* had larger caudate nucleus volumes in comparison to controls (Table 2). This main effect was further qualified by a IPV-by-sex interaction. In sex-stratified analyses, IPV exposed boys had 5% larger caudate volumes compared to unexposed boys (3852 vs. 3665 mm^3^; *p* = 0.008; Cohen’s *d* = −0.69 [95% CI −1.17 to −0.20]). In contrast, girls exposed to IPV did not differ from unexposed girls (3687 vs. 3708 mm^3^; *p* = .211; Cohen’s *d* = 0.06 [95% CI 0.41 to 0.53]). There was no main IPV effect on amygdala volume; however, a significant IPV-by-sex interaction was observed. Analyses stratified by sex demonstrated 2% smaller amygdala volumes in IPV-exposed compared to unexposed female infants (1069 vs. 1086 mm^3^; *p* = 0.028; Cohen’s *d* = 0.45 [95% CI −0.03 to 0.92]), whereas no significant difference was present in male infants (1096 vs. 1081 mm^3^; *p* = 0.283; Cohen’s *d* = −0.41 [95% CI −0.88 to 0.07]) (see Fig. 2A). Neither the amygdala nor caudate results survived correction for multiple comparisons. There was no evidence that antenatal IPV exposure was associated with the volume of the hippocampus, pallidum, putamen, or thalamus. Results from models unadjusted for covariates (Table S3) show effects of similar direction and magnitude.

**Table 2:** Adjusted associations between maternal IPV exposure and its interaction with neonatal sex on whole brain and subcortical grey matter volumes.

	IPV Mean (SD)	Control Mean (SD)	Unstandardized IPV β (SE)	IPV P value	Effect size, Cohen’s d (95% CI)	Unstandardized IPV × sex β (SE)	IPV × sex P value	Partial eta-squared (95% CI)
*Global*								
Total WM	119,994 (9528)	121,826 (11,748)	-1903 (2564)	0.459	0.17 (−0.16 to 0.50)	-881 (3639)	0.809	0.01 (0–0.06)
Total GM	239,034 (13,970)	235,802 (12,661)	4678 (3002)	0.122	-0.24 (−0.57 to 0.09)	-3759 (4261)	0.379	0.01 (0–0.06)
*Subcortical regions*								
Amygdala	1082 (42)	1083 (37)	9.51 (8.86)	0.258	0.03 (−0.30 to 0.36)	-28.36 (12.63)	0.027 *	0.04 (0–0.12)
Caudate	3765 (320)	3686 (314)	166.98 (73.28)	0.024 *	-0.25 (−0.58 to 0.08)	-213.65 (103.98)	0.042 *	0.03 (0–0.11)
Hippocampus	3210 (249)	3166 (235)	43.86 (54.22)	0.420	-0.19 (−0.52 to 0.15)	-59.24 (76.93)	0.443	0.01 (0–0.05)
Pallidum	1426 (58)	1422 (59)	13.50 (13.96)	0.335	-0.07 (−0.40 to 0.26)	-13.24 (19.81)	0.505	0.01 (0–0.05)
Putamen	5674 (162)	5679 (159)	49.06 (39.38)	0.215	0.03 (−0.30 to 0.36)	-104.00 (55.88)	0.065	0.03 (0–0.10)
Thalamus	6418 (190)	6420 (213)	27.03 (49.76)	0.588	0.01 (−0.32 to 0.34)	-70.33 (70.61)	0.321	0.09 (0–0.06)

IPV exposure (0 = below threshold, 1 = above threshold), infant sex (male = 0; female = 1) and their interaction, as predictors of neonatal structural brain volumes previously corrected for estimated total intracranial volume. All models are adjusted for household income, maternal HIV status, maternal depressive symptoms, antenatal smoking and alcohol consumption, duration of gestation, and infant birthweight. Positive IPV regression coefficients indicate that IPV exposure is associated with higher volumes for that region.

Notes: Volumes are sum of left and right hemispheres in mm^3^.

*Uncorrected *p* < 0.05.

Abbreviations: β, unstandardized beta coefficient; CI, confidence interval; IPV, *in utero* exposure to intimate partner violence.

### 3.3 Effects of antenatal IPV exposure on neonatal white matter microstructure

#### 3.3.1 Uncinate fasciculus

Infants who were exposed to IPV *in utero* had higher MD in the uncinate fasciculus compared to controls (Table 3), which was further qualified by an IPV-by-sex interaction. Analyses stratified by sex demonstrated that IPV exposure predicted 4% higher uncinate fasciculus MD in boys (2.00 vs. 1.92, *p* = 0.002; Cohen’s *d* = −0.94 [95% CI −1.64 to −0.23]), whereas no association was found in girls (1.92 vs. 1.93, *p* = 0.297; Cohen’s *d* = 0.13 [95% CI −0.57 to 0.82]). Both the main and interaction effects remained significant after multiple comparison correction. There were no significant associations between IPV or IPV-by-sex interactions on FA. Results from models unadjusted for covariates (Supplementary Table 4 [ST4]) show effects of similar magnitude and direction.

**Table 3:** Adjusted associations between maternal antenatal IPV exposure and its interaction with neonatal sex on microstructural measures for white matter tracts.

	IPV Mean (SD)	Control Mean (SD)	Unstandardized IPV β (SE)	IPV P value	Effect size, Cohen’s d (95% CI)	Unstandardized IPV × sex β (SE)	IPV × sex P value	Partial eta-squared (95% CI)
*Uncinate fasciculus*								
FA	0.274 (0.028)	0.282 (0.021)	-0.016 (0.008)	0.057	0.31 (−0.18 to 0.79)	0.014 (0.012)	0.241	0.02 (0–0.15)
MD	1.958 (0.094)	1.925 (0.088)	0.095 (0.029)	0.002 *^δ^	-0.37 (−0.85 to 0.12)	-0.124 (0.042)	0.004 *^δ^	0.13 (0.01–0.30)
*Fornix*								
FA	0.318 (0.028)	0.328 (0.022)	-0.016 (0.008)	0.064	0.41 (−0.09 to 0.90)	0.010 (0.012)	0.414	0.012 (0–0.121)
MD	1.923 (0.090)	1.906 (0.069)	0.043 (0.024)	0.078	-0.22 (−0.70 to 0.26)	-0.037 (0.035)	0.293	0.02 (0–0.13)
*Cingulum*								
FA	0.232 (0.024)	0.240 (0.024)	-0.010 (0.009)	0.273	0.33 (−0.15 to 0.81)	0.005 (0.013)	0.695	0.01 (0–0.08)
MD	1.773 (0.067)	1.757 (0.058)	0.018 (0.021)	0.387	-0.253 (−0.73 to 0.23)	0.004 (0.030)	0.898	0.01 (0–0.08)
*Corpus callosum*								
FA	0.299 (0.024)	0.311 (0.017)	-0.012 (0.008)	0.135	0.60 (0.11–1.09)	-0.002 (0.011)	0.846	0.01 (0–0.06)
MD	1.095 (0.042)	1.079 (0.038)	0.041 (0.013)	0.002 *^δ^	-0.42 (−0.90–0.06)	-0.049 (0.019)	0.010 *	0.11 (0.01–0.27)
*Corticospinal tract*								
FA	0.327 (0.036)	0.338 (0.032)	-0.034 (0.012)	0.008 *	0.32 (−0.16 to 0.81)	0.044 (0.018)	0.016 *	0.10 (0.01–0.26)
MD	1.580 (0.083)	1.558 (0.075)	0.043 (0.024)	0.074	-0.29 (−0.77 to 0.20)	-0.029 (0.034)	0.403	0.01 (0–0.12)

IPV exposure (0 =below threshold; 1 = above threshold), infant sex (male = 0; female = 1) and their interaction, as predictors of neonatal diffusion outcomes. All models are adjusted for household income, maternal HIV status, maternal depressive symptoms, antenatal smoking and alcohol consumption, duration of gestation, and infant birthweight. Positive IPV regression coefficients indicate that IPV exposure is associated with higher diffusion values for that region.

Notes: Diffusion coefficients are averaged across hemispheres (×10^-3^ mm^2^/s).

*Uncorrected *p* < 0.05.

^δ^DTI comparisons to survive multiple comparison correction using the false discovery rate across the 5 regions and 2 metrics, which generated a corrected overall *p*-value of 0.005.

Abbreviations: β, unstandardized beta coefficient; CI, confidence interval; IPV, *in utero* exposure to intimate partner violence; MD, mean diffusivity; FA, fractional anisotropy.

#### 3.3.2 Corpus callosum

Similarly, corpus callosum MD was higher in IPV-exposed infants compared to controls (Table 3). There was also a significant IPV-by-sex interaction; in sex-stratified analyses, IPV exposure predicted 4% higher corpus callosum MD in boys (1.12 vs. 1.08, *p* = 0.004; Cohen’s *d* = −0.90 [95% CI −1.60 to −0.20]). In contrast, IPV did not predict corpus callosum MD in girls (1.08 vs. 1.08, *p* = 0.389; Cohen’s *d* = 0.04 [95% CI −0.66 to 0.73]). The main effect survived FDR correction for multiple comparisons, although the interaction effect did not. There were no significant associations between IPV or IPV-by-sex interactions on FA.

#### 3.3.3 Corticospinal tract

Finally, corticospinal tract FA was lower in IPV-exposed infants compared to controls (Table 3) and was further qualified by a IPV-by-sex interaction. IPV exposure predicted 9% lower corticospinal tract FA in boys (0.312 vs. 0.342, *p* = 0.005; Cohen’s *d* = 0.97 [95% CI 0.26–1.67]), but not girls (0.340 vs. 0.331, *p* = 0.400; Cohen’s *d* = −0.27 [95% CI −0.97 to 0.44]). Neither the main effect nor the interaction effect remained significant after FDR correction. There were no significant associations between IPV or IPV-by-sex interactions on MD.

#### 3.3.4 Fornix and Cingulum

There were no significant effects of IPV or IPV-by-sex interactions on FA or MD in the fornix or cingulum.

### 3.4 Sensitivity analyses

Our findings were further supported by sensitivity analyses in which mother-infant pairs who had pregnancy complications including pre-eclampsia, gestational diabetes mellitus, pelvic inflammatory disease and/or high blood pressure were removed. Excluding these infants from the analysis (*n* = 11) did not meaningfully change the associations or effect sizes reported above (Supplementary Tables 5 and 6 [ST5 and ST6]).

## 4 Discussion

This prospective study of South African infants (aged 2–6 weeks) was a novel examination of subcortical brain volumes and white matter microstructure whose mothers had been exposed to IPV during pregnancy. We found tentative evidence for sex-specific effects of maternal antenatal IPV exposureon young infant subcortical volumes: IPV predicted a *smaller* amygdala among females but not males, and a *larger* caudate nucleus in males but not females. No main effects of IPV or IPV-by-sex interactions were observed for the hippocampus, or any other subcortical region. In a subsample (*n* = 70) with diffusion imaging data available, we found robust evidence that infants exposed to IPV *in utero* possessed higher mean diffusivity in the uncinate fasciculus and corpus callosum and lower fractional anisotropy in the corticospinal tract. Additional analyses suggest that these microstructural alterations also vary as a function of infant sex: diffusivity alterations were apparent in males but not females exposed to IPV. All the observed effects remained significant after excluding mother-infant dyads with pregnancy complications. Taken together, our results suggest that volumetric and microstructural brain alterations are observed in IPV-exposed infants shortly after birth, implying that they occur in the intrauterine environment.

Our observations of sex-specific effects of IPV exposure on neonatal amygdala volume builds on previous findings that have reported sex differences in amygdala volume in children and adolescents whose mother's were exposed to antenatal psychological distress. However, the direction of effect was contrary to our initial prediction; amygdala volume was *lower* in IPV-exposed females in comparison to their unexposed counterparts, which contrasts with previous findings of enlarged amygdala volumes reported in older female children and adolescents (Acosta et al., 2019, Buss et al., 2012, Jones et al., 2019, Wen et al., 2017). Interestingly, the pattern emerging from recent studies of neonatal amygdala structural connectivity in relation to higher maternal cortisol is one of enhanced maturation and greater connections in female neonates, and potentially reduced amygdala development in males (Graham et al., 2019, Stoye et al., 2020). Taken together, these results suggest a possible delay in amygdala development in very young infant girls exposed to IPV and possible ‘catch- up’ growth thereafter, with brain growth patterns implicated in a vast range of psychiatric and developmental conditions (Brouwer et al., 2022). Longitudinal studies of brain development in this cohort will be able to shed light on this hypothesis.

In terms of other subcortical structures, we found no evidence that antenatal IPV exposure was related to variations to hippocampal volume in neonates, in support of previous literature (Buss et al., 2012, Favaro et al., 2015, Marečková et al., 2018), and there were also no effects on the thalamus, putamen, or pallidum. However, the caudate nucleus was found to be 5% larger among males, but not females, exposed to IPV. To our knowledge, this is the first report of caudate nucleus volume being associated with prenatal stress exposure. Alongside a key role in executive functioning, sensory integration, and socio-emotional processing (Choi et al., 2022, Lanciego et al., 2012), caudate structure and function may have important neurodevelopmental implications. For example, larger caudate volumes have been related to impaired problem solving and increased impulsivity in children with autism (Voelbel et al., 2006) and have recently been identified as a marker of severe neurodevelopmental delay within the first 2 years of life (Hüls et al., 2022). Given that our findings did not survive strict correction for multiple comparison testing suggests future studies may select the amygdala and caudate as *a prior* regions of interest in examining the neurological consequences of l psychological distress during pregnancy.

Our examination of white matter microstructural integrity found that antenatal maternal IPV exposure was associated with higher MD in the uncinate fasciculus and corpus callosum, and lower FA in the corticospinal tract. Strikingly, only IPV-exposed boys, but not girls, showed changes in diffusivity in these tracts, with very large effect sizes (Cohen’s *d* = −0.90 and 0.97). Lower FA/higher MD during development generally points to reduced microstructural integrity, including disrupted glial proliferation and maturation (Yoshida et al., 2013), as well as increased brain water content and decreases in axon density (Lautarescu et al., 2020, Wimberger et al., 1995). However, the direction of effect can also depend on developmental stage (Tamnes et al., 2018). The uncinate fasciculus is part of the temporo-amygdala-orbitofrontal network, and diffusivity alterations within this tract have previously been associated with disorders that are more prevalent in males, such as conduct problems (Passamonti et al., 2012, Sarkar et al., 2013) and autism spectrum conditions (Catani et al., 2016), suggesting that these early microstructural alterations may have adverse developmental consequences. The present study also supports previous literature which documents an association between prenatal psychological distress and changes to the diffusion of two major white matter pathways: the corpus callosum and corticospinal tract (Borchers et al., 2021, Zwicker et al., 2013). Overall, our findings provide a picture of disrupted microstructural white matter integrity in tracts that are central to socioemotional functioning in association with IPV exposure during pregnancy, specifically in male infants.

Exposure to IPV is a particularly severe stressor, and intense or prolonged stress during pregnancy increases fetal exposure to stress biomarkers (e.g., cortisol and pro-inflammatory cytokines) which can alter the development of the nervous system (Goldstein et al., 2021). There are plausible biological mechanisms that can help explain how an infant’s biological sex may modify the effect of IPV on brain structure and connectivity, with sex chromosomes in the placenta likely to produce sex-specific transplacental signals to the developing brain (Bale, 2016, Carpenter et al., 2017). Male and female fetuses also have different patterns of glucocorticoid expression during development, which may indicate different windows of vulnerability to cortisol exposure (Owen and Matthews, 2003). Further examination of these sex-specific effects may be key to understanding the sex bias in neurodevelopment disorders.

The present study has several strengths including its prospective design and the fact that the mothers and infants were well-characterized from a sociodemographic perspective. Statistical adjustment for plausible confounders that may impact fetal brain development is essential to isolate the specific impact of IPV exposure, with influential prenatal factors often not reported comprehensively in infant neuroimaging studies (Pulli et al., 2019). These include intrauterine alcohol exposure (Archibald et al., 2001, Donald et al., 2015), HIV status (Wedderburn et al., 2022), smoking (Ekblad et al., 2015, El Marroun et al., 2014), and maternal depression (Barnett et al., 2021, Groenewold et al., 2022). The high rates of IPV exposure in this sample (44% of the sample) provide approximately equal group sizes meaning the cohort is well-suited for studying the impact of antenatal IPV. Crucially, scans were conducted at a median of 21 days of life, limiting exposure to postnatal environmental factors that may also contribute to neurodevelopment.

The study also had several limitations. First, the administered IPV questionnaire assessed the mother’s experiences of IPV over the preceding 12 months and is therefore not strictly specific to the gestational period. Therefore, we cannot rule out that some women may have been exposed to IPV prior to conception and not during pregnancy. Similarly, it is also possible that some women were exposed to IPV after the questionnaire was administered at the study visit (i.e., between 28 and 32 weeks and birth). Second, the categorization of two groups based on IPV exposure does not capture significant heterogeneity within the IPV-exposed group; for example, some mothers may experience severe and chronic IPV whereas others may have only just reached the threshold for inclusion. A minority of women (*n* = 11) reported exposure to all types of IPV (emotional, physical, and sexual) within the previous 12 months, whereas the majority of women were subjected to emotional abuse only. Nonetheless, our sample size limited the ability to examine the impact of exposure severity. Third, we were unable to pinpoint the exact timing of IPV during pregnancy, with several studies highlighting the importance of considering the developmental window of vulnerability and its unique effects on brain outcomes (Acosta et al., 2019, Mareckova et al., 2022). Fourth, a greater proportion of IPV-exposed women drank alcohol during pregnancy (27%) compared to the control group (11%). While we have adjusted for prenatal alcohol exposure in all models, we acknowledge that this is only a first-order linear approximation of alcohol effects on brain outcomes that may not fully capture the full extent of the association. Finally, only a sub-sample of neonates had diffusion imaging data available due to movement and technical artefacts (49% of the cohort with structural MRI), which minimized statistical power for detecting IPV-by-sex interactions on white matter microstructure. Moreover, while TBSS is regarded as a standard approach for group comparisons of diffusion properties (Smith et al., 2006), concerns have been raised with regards to anatomical inaccuracies in skeleton projections (Bach et al., 2014). However, we sought to minimize this issue by using a custom template as a target in the registration step (Keihaninejad et al., 2012) to enhance registration quality and anatomical specificity (Bach et al., 2014).

## 5 Conclusion

We show how maternal exposure to IPV during pregnancy is associated with sex-specific alterations in brain structure in young infants shortly after birth, indicating that severe maternal distress in the antenatal period may impact early brain development and maturation *in utero*IPV exposure is associated with a smaller amygdala in females, but not males, and a larger caudate nucleus in males, but not females. Both structures are crucial for emotion regulation and cognitive control. Moreover, alterations to the microstructural integrity of white matter tracts were evident in IPV-exposed male but not female infants. These results are further strengthened by robustness after excluding mothers and infants with pregnancy complications. Longitudinal follow up will examine whether these observations persist into later childhood and investigate how maternal IPV exposure may affect postnatal brain growth trajectories. These findings have important implications for understanding whether antenatal maternal stress via IPV exposure contributes to a sex bias in cognitive, emotional, and behavioural disorders often observed in childhood.

## Funding sources

The present analyses were funded by the UK Medical Research Council (grant MR/T002816/1). The Drakenstein Child Health Study was funded by the 10.13039/100000865Bill and Melinda Gates Foundation (OPP 1017641). Additional support for KAD, HJZ and DJS is from the 10.13039/501100001322South African Medical Research Council (SA MRC) and this work was partly funded by a grant from Carnegie Corporation of New York.

## Declaration of Competing Interest

The authors declare that they have no known competing financial interests or personal relationships that could have appeared to influence the work reported in this paper.

## Data Availability

Data cannot be shared publicly because of ethical conditions with which study investigators are obliged to comply. Access to the project data is restricted to nominated investigators approved by the University of Cape Town Human Research Ethics Committee, as per the consent document. Interested, qualified researchers may request to access this data by contacting the Drakenstein Child Health Study (via **lesley.workman@uct.ac.za**) to submit a formal data use request and ensure required ethical approval received prior to use.
