# Supplementary material for: Antenatal maternal intimate partner violence exposure is associated with sex-specific alterations in brain structure among young infants: Evidence from a South African birth cohort
Source: Dev Cogn Neurosci. 2023 Feb 6;60:101210. doi: 10.1016/j.dcn.2023.101210 (PMC9929680; doi:10.1016/j.dcn.2023.101210)
Supplement: Supplementary file 1 — Supplementary material. [file mmc1.docx]

**Supplementary Information**

**Antenatal maternal intimate partner violence exposure is associated with sex-specific alterations in brain structure among young infants: Evidence from a South African birth cohort**

Lucy V. Hiscox, Graeme Fairchild, Kirsten A. Donald, Nynke A. Groenewold, Nastassja Koen, Annerine Roos, Katherine L Narr, Marina Lawrence, Nadia Hoffman, Catherine J. Wedderburn, Whitney Barnett, Heather J. Zar, Dan J. Stein, and Sarah L. Halligan

**Correspondence to:** Lucy Hiscox, lh2235@bath.ac.uk

**Supplementary Methods:** Neuroimaging acquisition, processing, and data quality assessment.

**Supplementary Table S1:** Details on imaging outliers.

**Supplementary Table S2:** Sociodemographic and clinical characteristics of both mothers and infants according to IPV status (IPV exposed vs. controls) for the subsample with diffusion tensor imaging (DTI) data.

**Supplementary Table S3:** Unadjusted associations between maternal IPV exposure and its interaction with neonatal sex on whole brain and subcortical grey matter volumes.

**Supplementary Table S4:** Unadjusted associations between maternal antenatal IPV exposure and its interaction with neonatal sex on microstructural measures for white matter tracts obtained using diffusion tensor imaging.

**Supplementary Table S5:** Excluding mother-infant dyads with pregnancy complications (*n*=11). Adjusted associations between maternal IPV exposure and its interaction with neonatal sex on whole brain and subcortical grey matter volumes.

**Supplementary Table S6:** Excluding mother-infant dyads with pregnancy complications (*n*=11). Adjusted associations between maternal antenatal IPV exposure and its interaction with neonatal sex on microstructural measures for white matter tracts obtained using diffusion tensor imaging.

**Supplementary analysis code:** MATLAB code for estimated total intracranial volume (eTIV) correction and STATA regression models.

*Neuroimaging acquisition*

MR images were acquired at the Cape Universities Brain Imaging Centre (CUBIC), Tygerberg Hospital, Cape Town using a Siemens Magnetom 3T Allegra MRI scanner (Erlangen, Germany). Infants were fed, swaddled in a blanket, and encouraged to sleep. A qualified nurse or pediatrician remained in the scanner room with the infant at all times, and a pulse oximeter monitored pulse and oxygenation throughout the scan. Given the challenges of scanning neonates, a radiofrequency transmit/receive head coil was used with a wet clay inlay (40x40cm, 2cm thickness, standard sculpting clay) and voltage was decreased to optimize signal.

- Sagittal T2-weighted images were acquired using the following parameters: repetition time (TR) = 3500ms; echo time (TE) = 354 ms; FOV = 160 x 160 mm; in-plane resolution= 1.3 x 1.3 x 1.0mm, 128 slices. Sequence scan time was 5 minutes 41 seconds.
- Diffusion weighted images were collected using a spin-echo echo-planar imaging (EPI) sequence with images collected in both the anterior–posterior (A-P) and posterior–anterior (P-A) phase encoding directions to correct for field inhomogeneities. Parameters were as follows: 30 diffusion directions; FOV = 160 × 160 mm, *TR* = 7800 *ms*, *TE* = 91 *ms*, voxel size 1.8 × 1.8 × 2.0 mm^3^; b-value 1 of 0 s/mm^2^ and b-value 2 of 1000 s/mm^2^. Total diffusion scan time was 12 mins 54 s.

*Neuroimaging processing*

Images were first converted from DICOM to NIfTI format using the dcm2nii conversion tool. NIfTI images were then brain extracted using the FSL 5.0 brain extraction tool (BET) (Smith, 2002). Each scan was visually checked following the initial BET and additional custom thresholds were applied to further improve brain extraction.

Acquired structural T2-weighted images were processed using Statistical Parametric Mapping Software (SPM8) (www.fil.ion.ucl.ac.uk/spm/software/spm8) run in Matlab R2017B, using the University of North Carolina (UNC) custom infant T2 template in Montreal Neurological Institute (MNI) standard space (Shi *et al.*, 2011). Data from 95 infants (39 females, 56 males) were used to create this template and the associated tissue probability maps served as priors for segmentation. The UNC infant atlas has demonstrated improved performance when compared to manual segmentation as well as other comparable atlases (Brown *et al.*, 2014; Tzarouchi *et al.*, 2014). Images were first registered using normalized and mutual information and then spatially normalized with modulation by the Jacobian to the UNC neonate T2 template using standard settings (Ashburner *et al.*, 2012). Normalised images were then segmented into grey matter, white matter, and cerebrospinal fluid using tissue priors as per the UNC infant template, while applying very light bias regularisation and normalization with modulation, and the resulting probabilistic maps were saved for further analysis (Shi *et al.*, 2011).

Two researchers visually inspected the normalized images and segmented grey matter maps to check segmentation accuracy and alignment to the template. A third researcher reviewed the scans and made a final decision where there was disagreement over alignment quality. Images that failed normalization or segmentation were discarded. Masking and extraction were performed using the Masking toolbox. Outlier detection was performed using the ENIGMA protocol (http://enigma.ini.usc.edu/protocols/imaging-protocols/), to determine any values that were greater than 1.5 times the interquartile range. Any regions marked as a statistical outlier were reinspected to evaluate segmentation. Grey matter segmentations from 146 infants passed quality control.

Grey matter volumes were extracted for subcortical regions using the automated anatomical labelling atlas (Tzourio-Mazoyer *et al.*, 2002): amygdala, caudate, hippocampus, pallidum, putamen, and thalamus. Total grey matter, white matter and cerebrospinal fluid estimates were also extracted and summed to obtain total intracranial volume.

The first portion of the diffusion pipeline used TORTOISE (Tolerably Obsessive Registration and Tensor Optimization Indolent Software Ensemble) (Pierpaoli *et al.*, 2010). TORTOISE is suited to pediatric populations who are inclined to move by implementing comprehensive correction and greater anatomical registration ability compared to mainstream diffusion processing pipelines.

Raw diffusion-weighted images were quality checked for movement and technical artefacts before preprocessing, to ensure that at least 15 volumes per image were usable. The individual high-resolution structural images aligned to the UNC template (mentioned previously) were used as an anatomical reference image in TORTOISE. For reverse polarity data (A-P and P-A), each DWI acquisition was run though DiffPrep (Wu *et al.*, 2008) in TORTOISE, separately, for susceptibility distortion correction, motion correction, and eddy current correction. Gradient directions were rotated and adjusted according to the eddy current and motion correction. Following DiffPrep, the output images from both the A-P and P-A DWI acquisitions were then sent through Diffeomorphic Registration for Blip-Up Blip-Down Diffusion Imaging DR-BUDDI) in TORTOISE (Irfanoglu et al., 2015) for further EPI distortion and eddy current correction.

The Tract-based Spatial Statistics (TBSS) pipeline in FMRIB Software Library (FSL version 5) (Smith *et al.*, 2006) in combination with DTIFIT was used to fit a diffusion tensor model at each voxel. A representative custom template was created during the registration step of TBSS, utilizing data from our cohort. This approach has been recommended for children and for studies with smaller sample sizes (Bach et al., 2014; Smith et al., 2006). This template was registered to FSL’s standard FMRIB58_FA (fractional anisotropy) image as an intermediate step, and individual images were linearly registered into standard MNI space using FLIRT (Jenkinson et al., 2002) by applying the same transforms calculated by registering the template to the FMRIB58_FA image. All individual images were then merged onto an average FA image for use as the registration target and a mean FA image and skeleton was created and subsequently thresholded at 0.15.

After skeletonization, ROI masks of the mean FA skeleton were then created for all 48 white matter tract regions as per the JHU ICBM-DTI-81 atlas (Mori *et al.*, 2008) and the mean value for each of the 48 regions were extracted from template space, using standard TBSS procedures, for further analysis. Mean diffusivity (MD) images were also derived from the mean FA skeleton and ROIs extracted for the 48 regions. In the study, we analyzed only 8 out of the 48 regions available (right and left uncinate fasciculus, fornix, right and left cingulum, body of the corpus callosum, and right and left corticospinal tract). The right and left of the uncinate fasciculus, cingulum and corticospinal tract were averaged together to provide bilateral measurements.

**Table S1:** Imaging outliers

| **Brain region** | **Shapiro-Wilk W test prior to the removal of imaging outliers (*p* value)** | **Number of outliers** | **Cook’s *d*** | **Shapiro-Wilk W test after the removal of imaging outliers (*p* value)** |
| --- | --- | --- | --- | --- |
| Amygdala | 0.0035 | 2 | 0.084 and 0.087 | 0.380 |
| Uncinate fasciculus FA | <0.0001 | 1 | 0.772 | 0.145 |
| Fornix FA | <0.0001 | 3 | 0.753 and 0.148 and 0.152 | 0.060 |
| Cingulum FA | <0.0001 | 2 | 0.763 and 0.073 | 0.189 |
| Corpus Callosum FA | <0.0001 | 2 | 0.754 and 0.102 | 0.010 |
| Corticospinal tract FA | <0.0001 | 1 | 0.738 | 0.139 |

Note: Any structure or metric not mentioned passed the assumptions for performing linear regression, and therefore no observations were removed.

**Table S2:** Sociodemographic and clinical information for mother and infant according to mother’s recent IPV exposure for the subsample of infants with usable DTI data.

|  | IPV-exposed  (*n* = 28) | | Controls  (*n* = 42) | Group differences | *p*-value |
| --- | --- | --- | --- | --- | --- |
| **Sociodemographics** |  |  | |  |  |
| **Clinic (*n*, %)**  *Mbekweni*  *TC Newman* | 10, 35.7%  18, 64.3% | 20, 47.6%  22, 52.4% | | *χ* ^2^ = 0.972 | 0.324 |
| Maternal age at birth *(M±SD)* | 27.67 ± 5.96 | 26.13 ± 6.04 | | *t* = -1.06 | 0.295 |
| **Household income per month (*n*, %)**  *<R1000*  *R1000 – R5000*  *>R5000* | 9, 32.1%  15, 53.6%  4, 14.3% | 14, 33.3%  21, 50.0%  7, 16.7% | | *χ* ^2^ (2) = 0.110 | 0.947 |
| **Employment status (n, %)**  *Employed*  *Unemployed* | 7, 25.0%  21, 75.0% | 17, 40.5%  25, 59.5% | | *χ* ^2^ = 1.79 | 0.181 |
| **Maternal education (*n*, %)**  *Primary/some secondary*  *Completed secondary/any tertiary* | 15, 53.6%  13, 46.4% | 20, 47.6%  22, 52.4% | | *χ* ^2^ = 0.238 | 0.626 |
| **Marital status (*n*, %)**  *Single (never married)*  *Married/living with partner* | 13, 46.4%  15, 53.6% | 29, 69.0%  13, 31.0% | | *χ* ^2^ = 3.58 | 0.058 |
| **Maternal clinical characteristics** |  |  | |  |  |
| **HIV status (*n*, %)**  *Positive*  *Negative* | 5, 17.9%  23, 82.1% | 10, 23.8%  32, 76.2% | | *χ* ^2^ = 0.354 | 0.552 |
| **Beck Depression Inventory (*n*, %)**  *Probable moderate/severe*  *Probable sub-threshold* | 8, 28.6%  20, 71.4% | 8, 19.0%  34, 81.0% | | *χ* ^2^ = 0.864 | 0.353 |
| **Prenatal alcohol exposure (*n*, %)**  *Exposure*  *No exposure* | 7, 25.0%  21, 75.0% | 7, 16.7%  35, 83.3% | | *χ* ^2^ = 0.729 | 0.393 |
| **Prenatal cotinine (*n*, %)** |  |  | |  |  |
| *Non-smoker (<10 ng/ml)*  *Passive smoker (>=10-499 ng/ml)*  *Active smoker (>=500 ng/ml)* | 5, 17.9%  7, 25.0%  16, 57.1% | 12, 28.6%  18, 42.9%  12, 28.6% | | *χ* ^2^ (2) = 5.723 | 0.057 |
| **Infant characteristics** |  |  | |  |  |
| **Sex (*n*, %)**  *Male*  *Female* | 13, 46.4%  15, 53.6% | 25, 59.5%  17, 40.5% | | *χ* ^2^ = 1.161 | 0.281 |
| Age at scan (weeks) (*M*±*SD*) | 3.21 ± 1.00 | 2.99 ± 0.73 | | *t* = -1.10 | 0.276 |
| Gestation age (weeks) (*M*±*SD*) | 38.93 ± 1.98 | 39.24 ± 1.46 | | *t* = 0.752 | 0.455 |
| Birthweight (grams) (*M*±*SD*) | 3045 ± 500 | 3229 ± 386 | | *t* = 1.73 | 0.089 |
| Head circumference (cm) (*M*±*SD*) | 33.48 ± 1.70 | 34.02 ± 1.60 | | *t* = 1.354 | 0.180 |

**Table S3.**  Unadjusted associations between maternal IPV exposure and its interaction with neonatal sex on whole brain and subcortical grey matter volumes.

|  | IPV Mean (SD) | Control Mean (SD) | Unstandardized IPV *β (SE)* | IPV  *P value* | Effect size, Cohen’s d (95% CI) | Unstandardized IPV × infant sex *β* (*SE*) | IPV × infant sex *P value* | Partial eta-squared (95% CI) |
| --- | --- | --- | --- | --- | --- | --- | --- | --- |
| *Global* | | | | | | | | |
| Total WM | 119,994  (9528) | 121,826  (11,748) | -700  (2573) | 0.786 | 0.17  (-0.16 to 0.50) | -2565  (3630) | 0.481 | 0.004  (0 to 0.048) |
| Total GM | 239,033  (13,970) | 235,801  (12,661) | 4946  (2987) | 0.100 | -0.24  (-0.57 to 0.09) | -2905  (4359) | 0.506 | 0.003  (0 to 0.043) |
| *Subcortical regions* | | | | | | | | |
| Amygdala | 1082  (42) | 1083  (37) | 15.83  (9.25) | 0.089 | 0.03  (-0.31 to 0.36) | -33.05  (13.06) | 0.013* | 0.045  (0.002 to 0.128) |
| Caudate nucleus | 3765  (320) | 3686  (314) | 186.64  (75.00) | 0.014* | -0.25  (-0.58 to 0.08) | -208.10  (105.83) | 0.051 | 0.027  (0 to 0.099) |
| Hippocampus | 3210  (249) | 3166  (235) | 93.31  (57.58) | 0.107 | -0.19  (-0.52 to 0.15) | -92.97  (81.25) | 0.254 | 0.009  (0 to 0.064) |
| Pallidum | 1426  (58) | 1422  (59) | 13.63  (13.89) | 0.328 | -0.07  (-0.40 to 0.26) | -17.06  (19.60) | 0.386 | 0.005  (0 to 0.054) |
| Putamen | 5674  (162) | 5679  (159) | 43.80  (38.04) | 0.251 | 0.03  (-0.30 to 0.36) | -96.20  (53.67) | 0.075 | 0.023  (0 to 0.091) |
| Thalamus | 6418  (190) | 6420  (213) | 43.43  (48.67) | 0.374 | 0.01  (-0.32 to 0.34) | -88.69  (68.67) | 0.199 | 0.012  (0 to 0.069) |

IPV exposure (0 = below threshold, 1 = above threshold), infant sex (male = 0; female = 1) and their interaction, as predictors of neonatal structural brain volumes previously corrected for estimated total intracranial volume. Positive IPV regression coefficients indicate that IPV exposure is associated with higher volumes for that region.

Notes: Volumes are sum of left and right hemispheres in mm^3^.

*Uncorrected *p* < 0.05.

Abbreviations: β, unstandardized beta coefficient; CI, confidence interval; IPV, *in utero* exposure to intimate partner violence.

**Table S4**. Unadjusted associations between maternal antenatal IPV exposure and its interaction with neonatal sex on microstructural measures for white matter tracts

|  | IPV Mean (SD) | Control Mean (SD) | Unstandardized IPV *β (SE)* | IPV  *P value* | Effect size, Cohen’s d (95% CI) | Unstandardized IPV × infant sex *β* (*SE*) | IPV × infant sex *P value* | Partial eta-squared (95% CI) |
| --- | --- | --- | --- | --- | --- | --- | --- | --- |
| *Uncinate fasciculus* | | | | | | | | |
| FA | 0.274  (0.028) | 0.282  (0.021) | -0.013  (0.008) | 0.124 | 0.31  (-0.18 to 0.79) | 0.010  (0.012) | 0.406 | 0.011  (0 to 0.105) |
| MD | 1.958  (0.094) | 1.925 (0.088) | 0.080  (0.030) | 0.010* | -0.37  (-0.85 to 0.12) | -0.091  (0.043) | 0.039* | 0.063  (0 to 0.198) |
| *Fornix* | | | | | | | | |
| FA | 0.318  (0.028) | 0.328  (0.022) | -0.013  (0.009) | 0.125 | 0.41  (-0.09 to 0.90) | 0.007  (0.013) | 0.575 | 0.010  (0 to 0.122) |
| MD | 1.923  (0.090) | 1.906  (0.069) | 0.034  (0.026) | 0.203 | -0.22  (-0.70 to 0.26) | -0.025  (0.038) | 0.504 | 0.007  (0 to 0.092) |
| *Cingulum* | | | | | | | | |
| FA | 0.232  (0.024) | 0.240  (0.024) | -0.010  (0.008) | 0.259 | 0.35  (-0.14 to 0.83) | 0.003  (0.012) | 0.816 | 0.001  (0 to 0.058) |
| MD | 1.773  (0.067) | 1.757  (0.058) | 0.007  (0.021) | 0.742 | -0.25  (-0.73 to 0.23) | 0.021  (0.031) | 0.491 | 0.007  (0 to 0.093) |
| *Corpus callosum* | | | | | | | | |
| FA | 0.299  (0.024) | 0.311  (0.017) | -0.010  (0.007) | 0.138 | 0.60  (0.11 to 1.09) | -0.004  (0.010) | 0.741 | 0.002  (0 to 0.068) |
| MD | 1.095  (0.042) | 1.079  (0.038) | 0.037  (0.013) | 0.007* | -0.42  (-0.90 to 0.06) | -0.038  (0.019) | 0.049* | 0.057  (0 to 0.189) |
| *Corticospinal tract* | | | | | | | | |
| FA | 0.327  (0.036) | 0.338  (0.032) | -0.030  (0.011) | 0.008* | 0.32  (-0.16 to 0.81) | 0.039  (0.016) | 0.017* | 0.085  (0.002 to 0.228) |
| MD | 1.580  (0.083) | 1.558  (0.075) | 0.017  (0.027) | 0.522 | -0.29  (-0.76 to 0.20) | 0.014  (0.039) | 0.730 | 0.002  (0 to 0.068) |

IPV exposure (0 = below threshold, 1 = above threshold), infant sex (male = 0; female = 1) and their interaction, as predictors of diffusion outcomes. Positive IPV regression coefficients indicate that IPV exposure is associated with higher diffusion values for that region.

Notes: Diffusion coefficients are averaged across hemispheres (MD = ×10^-3^ mm^2^/s).

*Uncorrected *p* < 0.05.

^δ^DTI comparisons to survive multiple comparison correction using the false discovery rate across the 5 regions and 2 metrics, which generated a corrected overall *p*-value of 0.005.

Abbreviations: β, unstandardized beta coefficient; CI, confidence interval; IPV, *in utero* exposure to intimate partner violence; ns, non-significant; MD, mean diffusivity; FA, fractional anisotropy.

**Table S5**. Excluding mother-infant dyads with pregnancy complications (*n*=11). Adjusted associations between maternal IPV exposure and its interaction with neonatal sex on whole brain and subcortical grey matter volumes.

|  | IPV Mean (SD) | Control Mean (SD) | Unstandardized IPV *β (SE)* | IPV  *P value* | Effect size, Cohen’s d (95% CI) | Unstandardized IPV × infant sex *β* (*SE*) | IPV × infant sex *P value* | | Partial eta-squared (95% CI) | |
| --- | --- | --- | --- | --- | --- | --- | --- | --- | --- | --- |
| *Global* | | | | | | | | | |  |
| Total WM | 120,138  (9858) | 121,554  (11668) | -1199  (2724) | 0.661 | 0.13  (-0.22 to 0.47) | -1336  (3850) | 0.729 | 0.01  (0 to 0.04) | |  |
| Total GM | 238,719  (14170) | 236,706  (12017) | 3470  (3085) | 0.263 | -0.16  (-0.50 to 0.19) | -2905  (4359) | 0.506 | 0.01  (0 to 0.05) | |  |
| *Subcortical regions* | | | | | | | | | |  |
| Amygdala | 1080  (40) | 1083  (37) | 7.84  (9.23) | 0.397 | 0.07  (-0.28 to 0.41) | -23.83  (13.09) | 0.071 | 0.03  (0 to 0.11) | |  |
| Caudate nucleus | 3765  (324) | 3691  (314) | 135.67  (77.90) | 0.084 | -0.23  (-0.58 to 0.11) | -182.76  (110.1) | 0.099 | 0.02  (0 to 0.10) | |  |
| Hippocampus | 3188  (246) | 3171  (234) | 21.09  (57.88) | 0.716 | -0.07  (-0.42 to 0.27) | -31.37  (81.79) | 0.702 | 0.01  (0 to 0.04) | |  |
| Pallidum | 1426  (52) | 1422  (58) | 12.02  (14.14) | 0.397 | -0.06  (-0.41 to 0.28) | -8.34  (19.99) | 0.677 | 0.01  (0 to 0.04) | |  |
| Putamen | 5681  (162) | 5682  (152) | 52.28  (40.50) | 0.199 | 0.01  (-0.34 to 0.35) | -104.45  (57.23) | 0.070 | 0.03  (0 to 0.11) | |  |
| Thalamus | 6410  (177) | 6419  (216) | 26.52  (52.05) | 0.611 | 0.05  (-0.30 to 0.39) | -70.60  (73.57) | 0.339 | 0.01  (0 to 0.07) | |  |

IPV exposure (0 = below threshold, 1 = above threshold), infant sex (male = 0; female = 1) and their interaction, as predictors of neonatal structural brain volumes previously corrected for estimated total intracranial volume. All models are adjusted for household income, maternal HIV status, maternal depressive symptoms, antenatal smoking alcohol consumption, duration of gestation, and birthweight. Positive IPV regression coefficients indicate that IPV exposure is associated with higher volumes for that region.

Notes: Volumes are sum of left and right hemispheres in mm^3^.

*Uncorrected *p* < 0.05.

Abbreviations: β, unstandardized beta coefficient; CI, confidence interval; IPV, *in utero* exposure to intimate partner violence

**Table S6**. Excluding mother-infant dyads with pregnancy complications (*n*=11). Adjusted associations between maternal antenatal IPV exposure and its interaction with neonatal sex on microstructural measures for white matter tracts obtained using diffusion tensor imaging.

|  | IPV Mean (SD) | Control Mean (SD) | Unstandardized IPV *β (SE)* | IPV  *P value* | Effect size, Cohen’s d (95% CI) | Unstandardized IPV × sex *β* (*SE*) | IPV × sex *P value* | Partial eta-squared (95% CI) |
| --- | --- | --- | --- | --- | --- | --- | --- | --- |
| *Uncinate fasciculus* | | | | | | | | |
| FA | 0.275  (0.027) | 0.281  (0.022) | -0.014  (0.009) | 0.126 | 0.26  (-0.25 to 0.76) | 0.009  (0.013) | 0.453 | 0.01  (0 to 0.12) |
| MD | 1.957  (0.085) | 1.929 (0.086) | 0.094  (0.030) | 0.003*^δ^ | -0.32  (-0.83 to 0.18) | -0.116  (0.043) | 0.010* | 0.12  (0.01 to 0.29) |
| *Fornix* | | | | | | | | |
| FA | 0.321  (0.027) | 0.329  (0.022) | -0.015  (0.009) | 0.104 | 0.32  (-0.20 to 0.84) | 0.009  (0.013) | 0.501 | 0.010  (0 to 0.122) |
| MD | 1.916  (0.086) | 1.907  (0.069) | 0.045  (0.026) | 0.094 | -0.12  (-0.63 to 0.38) | -0.037  (0.037) | 0.323 | 0.02  (0 to 0.14) |
| *Cingulum* | | | | | | | | |
| FA | 0.233  (0.025) | 0.240  (0.024) | -0.102  (0.010) | 0.316 | 0.30  (-0.21 to 0.81) | 0.004  (0.014) | 0.794 | 0.01  (0 to 0.08) |
| MD | 1.769  (0.067) | 1.755  (0.059) | 0.020  (0.023) | 0.394 | -0.22  (-0.72 to 0.28) | 0.009  (0.032) | 0.783 | 0.01  (0 to 0.08) |
| *Corpus callosum* | | | | | | | | |
| FA | 0.300  (0.025) | 0.311  (0.015) | -0.011  (0.008) | 0.170 | 0.59  (0.07 to 1.10) | -0.005  (0.011) | 0.681 | 0.003  (0 to 0.096) |
| MD | 1.092  (0.039) | 1.080  (0.036) | 0.040  (0.013) | 0.004*^δ^ | -0.31  (-0.82 to 0.19) | -0.048  (0.019) | 0.013* | 0.114  (0.005 to 0.283) |
| *Corticospinal tract* | | | | | | | | |
| FA | 0.328  (0.036) | 0.339  (0.032) | -0.034  (0.013) | 0.012* | 0.31  (-0.20 to 0.82) | 0.443  (0.019) | 0.022* | 0.100  (0.001 to 0.268) |
| MD | 1.574  (0.077) | 1.562  (0.073) | 0.036  (0.024) | 0.152 | -0.16  (-0.66 to 0.35) | -0.015  (0.035) | 0.662 | 0.004  (0 to 0.095) |

IPV exposure (0 = below threshold, 1 = above threshold), infant sex (male = 0; female = 1) and their interaction, as predictors of diffusion outcomes. All models are adjusted for household income, maternal HIV status, maternal depressive symptoms, antenatal smoking alcohol consumption, duration of gestation, and birthweight. Positive IPV regression coefficients indicate that IPV exposure is associated with higher diffusion values for that region.

Notes: Diffusion coefficients are averaged across hemispheres (×10^-3^ mm^2^/s).

*Uncorrected *p* < 0.05.

^δ^DTI comparisons to survive multiple comparison correction using the false discovery rate across the 5 regions and 2 metrics, which generated a corrected overall *p*-value of 0.005.

Abbreviations: β, unstandardized beta coefficient; CI, confidence interval; IPV, *in utero* exposure to intimate partner violence; ns, non-significant; MD, mean diffusivity; FA, fractional anisotropy

Analysis code

// Correct for estimated total intracranial volume (eTIV)

function [ Normarray ] = NormalizeROI( icv_col,start_col,end_col,table)

if nargin < 4

disp('Must specify four arguments.');

return

end

ICVarray = table(:,icv_col);

meanICV = mean(ICVarray);

if end_col < start_col

disp('The start column cannot be greater than the end column.');

return

end

for j=start_col:end_col

ROIarray = table(:,j);

coeff = regstats(ROIarray,ICVarray,'linear','beta');

[x,y] = size(table);

betaarray(j,1) = coeff.beta(2,1);

for i=1:x

normalized = table(i,j) - ( coeff.beta(2,1) * ( table(i,icv_col) - meanICV ) );

if start_col ~= 1

minus = 1 - start_col;

newj = (j + minus);

Normarray(i,newj) = normalized;

else

Normarray(i,j) = normalized;

end

end

end

// Check regression assumptions for normality and multicollinearity

predict r, rstudent

iqr r

swilk r

estat imtest

estat hettest

// Outliers

predict d, cooksd

list brain_region ipv_any_recent child_sex shortpid d if d>4/N

// Regression models

regress brain_region ib0.ipv_any_recent##ib0.child_sex i.household_income i.maternal_hiv i.bdi_total_threshold c.birthweight c.gestation_age i.prenatal_cotinine i.prenatal_alcohol_composite

// Sex-stratified models

Bysort child_sex: regress brain_region ib0.ipv_any_recent i.household_income i.maternal_hiv i.bdi_total_threshold c.birthweight c.gestation_age i.prenatal_cotinine i.prenatal_alcohol_composite

// mean and SD

univar brain_region, by(ipv_any_recent)

// Cohen's d

esize twosample brain_region, by(ipv_any_recent) all

//partial eta squared

estat esize

References

Ashburner, J., Barnes, G., Chen, C., Daunizeau, J., Flandin, G., Friston, K., . . . Litvak, V. (2012). SPM8 manual. *Functional Imaging Laboratory, Institute of Neurology*.

Brown, C. J., Miller, S. P., Booth, B. G., Andrews, S., Chau, V., Poskitt, K. J., & Hamarneh, G. (2014). Structural network analysis of brain development in young preterm neonates. *NeuroImage, 101*, 667-680. doi:10.1016/j.neuroimage.2014.07.030

Mori, S., Oishi, K., Jiang, H., Jiang, L., Li, X., Akhter, K., . . . Mazziotta, J. (2008). Stereotaxic white matter atlas based on diffusion tensor imaging in an ICBM template. *NeuroImage, 40*(2), 570-582. doi:<https://doi.org/10.1016/j.neuroimage.2007.12.035>

Pierpaoli, C., Walker, L., Irfanoglu, M. O., Barnett, A., Basser, P., Chang, L.-C., . . . Sarlls, J. (2010). *TORTOISE: an integrated software package for processing of diffusion MRI data.* Paper presented at the ISMRM 18th annual meeting.

Shi, F., Yap, P.-T., Wu, G., Jia, H., Gilmore, J. H., Lin, W., & Shen, D. (2011). Infant Brain Atlases from Neonates to 1- and 2-Year-Olds. *PLOS ONE, 6*(4), e18746. doi:10.1371/journal.pone.0018746

Smith, S. M. (2002). Fast robust automated brain extraction. *Human brain mapping, 17*(3), 143-155. doi:10.1002/hbm.10062

Smith, S. M., Jenkinson, M., Johansen-Berg, H., Rueckert, D., Nichols, T. E., Mackay, C. E., . . . Behrens, T. E. (2006). Tract-based spatial statistics: voxelwise analysis of multi-subject diffusion data. *NeuroImage, 31*(4), 1487-1505. doi:10.1016/j.neuroimage.2006.02.024

Tzarouchi, L. C., Drougia, A., Zikou, A., Kosta, P., Astrakas, L. G., Andronikou, S., & Argyropoulou, M. I. (2014). Body growth and brain development in premature babies: an MRI study. *Pediatric radiology, 44*(3), 297-304. doi:10.1007/s00247-013-2822-y

Tzourio-Mazoyer, N., Landeau, B., Papathanassiou, D., Crivello, F., Etard, O., Delcroix, N., . . . Joliot, M. (2002). Automated Anatomical Labeling of Activations in SPM Using a Macroscopic Anatomical Parcellation of the MNI MRI Single-Subject Brain. *NeuroImage, 15*(1), 273-289. doi:<https://doi.org/10.1006/nimg.2001.0978>

Wu, M., Chang, L.-C., Walker, L., Lemaitre, H., Barnett, A. S., Marenco, S., & Pierpaoli, C. (2008). *Comparison of EPI distortion correction methods in diffusion tensor MRI using a novel framework.* Paper presented at the International Conference on Medical Image Computing and Computer-Assisted Intervention.
